# Effect of CIK on multidrug-resistance reversal and increasing the sensitivity of ADR in K562/ADR cells

**DOI:** 10.3892/ol.2014.2337

**Published:** 2014-07-10

**Authors:** LEI WANG, QI DENG, JIAN WANG, XUE BAI, XIA XIAO, HAI-RONG LV, MING-FENG ZHAO, PENG-JIANG LIU

**Affiliations:** 1 International Medical Center, The First Central Hospital of Tianjin, Tianjin 300192, P.R. China; 2 Department of Hematology, The First Central Hospital of Tianjin, Tianjin 300192, P.R. China

**Keywords:** cytokine-induced killer, Adriamycin, K562/ADR cells, multidrug-resistance

## Abstract

Leukemia is a leading cause of cancer-related mortality in children worldwide, and multidrug-resistance (MDR) is a main reason for tumor chemotherapy failure. The present study investigated the effects of ADR following incubation with cytokine-induced killer (CIK) cells on reversing MDR in K562/ADR cells. Mononuclear cells were isolated from the peripheral blood of healthy individuals and cultured *in vitro* in the presence of a combination of cytokines to generate CIK for K562/ADR cell treatment. A decreased level of P-glycoprotein expression and glutathione (GSH), an increased intracellular Rh-123 content, decreased mRNA and protein expression levels of MDR gene 1, MDR-associated protein 1, GSH S-transferase-π, B-cell lymphoma 2 and Survivin, and the decreased phosphorylation of AKT and the transcriptional activity of nuclear factor-κB and activator protein 1 were detected following ADR treatment in CIK co-cultured K562/ADR cells. Additionally, the level of ADR sensitivity and the apoptosis rate were increased in the CIK co-cultured K562/ADR cells. These results indicate that pre-treatment with CIK could reverse the MDR of K562/ADR cells, and that patients would be most likely to benefit from the combination of chemotherapy and CIK therapy.

## Introduction

Multidrug-resistance (MDR) is the major complication and a formidable obstacle in the therapy of acute leukemia (AL). Although allogeneic hematopoietic stem cell transplantation (HSCT) is a highly effective treatment for leukemia, its therapeutic potential is counterbalanced by treatment-related toxicity and graft-versus-host disease (GVHD). Therefore, the development of therapy to reverse MDR is extremely important in the therapy of AL (1,2).

Immunotherapy, which stimulates a patient’s own immune system, is a promising method to overcome the drug resistance to chemotherapy. Cytokine-induced killer (CIK) cells are major histocompatibility complex (MHC)-unrestricted cytotoxic lymphocytes generated with tumor necrosis factor-α (TNF-α), interferon-γ (IFN-γ), interleukin (IL)-2 and IL-12. The activity of CIK cells with regard to tumor cells is effective, moderate and MHC-unrestricted. This activity is mainly associated with the high proliferation potential of CIK cells (3,4)

In the present study, CIK cells were obtained from the peripheral blood of healthy donors. The K562/ADR cells were cultured with Adriamycin following incubation with CIK cells. The study analyzed the effects of ADR following incubation with CIK on reversing MDR in the K562/ADR cells and clarified its mechanism.

## Materials and methods

### Cell culture

Human cell lines (K562/ADR and K562) were preserved by tje International Medical Center of the First Central Hospital of Tianjin (Tianjin, China) and cultured in RPMI-1640 complete medium (Cambrex Bio Science, Verviers, Belgium) containing 10% heat inactivated fetal calf serum (FCS), 100 U/ml penicillin and 100 mg/ml streptomycin. Prior to the study, the K562/ADR cells were cultured in complete culture solution without Adriamycin.

### Generation of CIK cells

Human peripheral blood mononuclear cells were isolated from six healthy donors by Ficoll-Paque density centrifugation (GE Healthcare, Fairfield, CT, USA) at 500 × g for 20 min and washed three times with phosphate-buffered saline (PBS). The final cells were resuspended at a density of 3×10^6^ cells/ml in RPMI-1640 complete medium containing 10% heat inactivated FCS, 100 U/ml penicillin and 100 mg/ml streptomycin, and were seeded at 37°C, 5% CO_2_. To generate CIK cells, IFN-γ (1×10^6^ U/l) was added on day 1 and rhIL-1 (1×10^5^ U/l) and rhIL-2 (1×10^6^ U/l) were added on day 2. Fresh complete medium with rhIL-2 (1×10^6^ U/l) was added every 2–3 days, and the cells were harvested on day 14.

### Cytokine secretion assays

Cytokine secretion by the CIKs was detected by enzyme-linked immunosorbent assay (ELISA; R&D Systems, Emeryville, CA, USA) where 3×10^5^ cells were seeded into a 6-well microplate and incubated overnight. The medium without FCS was added, and the secretion of TNF-α, IFN-γ, IL-2 and IL-12 was measured 72 h later following the manufacturer’s instructions.

### Cytotoxicity assays

MTS cytotoxicity assays were used to determine the viability and proliferation of the K562/ADR cells. The K562/ADR cells were divided into five groups: K562 cells (Parent group), K562/ADR cells (Group I), K562/ADR with CIK (Group II) and K562/ADR with ADR in combination with CIK (Group III). The effect (CIK)/target (K562/ADR) ratio (E/T ratio) was 10:1 and 20:1 in groups II and III. The concentration of ADR was 0, 0.1, 0.5, 1, 5 and 10 μg/ml in the parent group, group I and group III. The cells of all groups were seeded at a density of 5×10^4^ cells/ml in 96-well plates with RPMI-1640 complete medium (100 μl/well) at 37°C in a 5% CO_2_ humidified atmosphere. Next, different E/T ratios (10:1 or 20:1) or ADR was added for 72 h. The cells were incubated for 2 h at 37°C, 5% CO_2_ with MTS agent (Promega Corporation, Madison, WI, USA) and the cytotoxicity of the K562/ADR and K562 cells was measured at 570 nm.

### Flow cytometry assays

The cells were cultured in a 6-well plate for 24 h, then incubated with different ratios of CIK (10:1 or 20:1) for 72 h. The cells were then digested, resuspended, incubated with P-gp antibodies for 30 min at 4°C and washed twice in PBS. The fluorescence intensity of fluorescein isothiocyanate (FITC)-P-gp (Abcam, Burlingame, CA, USA) was analyzed by flow cytometry (FACSCalibur; BD Biosciences, Franklin Lakes, NJ, USA) at 488 nm.

The cells were cultured in a 6-well plate for 24 h, then incubated with different ratios of CIK for 72 h. The cells were then digested, resuspended, incubated with 10 μM Rh-123 (Sigma-Aldrich, San Francisco, CA, USA) for 60 min and washed twice in PBS. The fluorescence intensity of Rh-123 was analyzed by flow cytometry (FACSCalibur; BD Biosciences) at 488 nm.

The cells were cultured in a 6-well plate for 24 h, then incubated with different ratios of CIK (10:1 or 20:1) for 72 h. A total of 10 μg/ml ADR was added and co-cultured for 24 h, then the cells were digested, resuspended, incubated with Annexin V-FITC and propidium iodide (PI) for 15 min at 37°C and washed twice in PBS. The apoptosis rate was analyzed by flow cytometry (FACSCalibur; BD Biosciences) at 488 nm.

### GSH determination assays

The cells were cultured in a 6-well plate for 24 h, then incubated with different ratios of CIK (10:1 or 20:1) for 72 h. Next, the cells were digested, resuspended and lysed. Intracellular GSH was measured the by the Total GSH Assay kit (Beyotime Institute of Biotechnology, Shanghai, China), according to the manufacturer’s instructions, using a Spectra Max M5 microplate reader (Molecular Devices Corporation, Sunnyvale, CA, USA).

### Western blotting assays

The cells were cultured in a 6-well plate for 24 h, then incubated with different ratios of CIK (10:1 or 20:1) for 72 h. The cells were then digested, resuspended and lysed. Next, centrifugation at 10,000 × g was performed for 10 min at 4°C, and the supernatant was extracted to obtain the total protein. Electrophoresis was performed in 12% SDS polyacrylamide gel and the protein was transferred to polyvinylidene fluoride membranes. The membranes were blocked by 5% skimmed milk overnight at 4°C, then monoclonal rabbit anti-human MDR1, MRP1, GST-π, Bcl-2, Survivin, p-AKT and β-actin antibodies (Santa Cruz Biotechnology, Inc., Santa Cruz, CA, USA) were added and incubated at 4°C overnight. The membranes were washed and incubated for 1 h with peroxidase-labeled anti-rabbit immunoglobulin G. Finally, the membranes were exposed to the Immobilon™ western chemiluminescent horseradish peroxidase substrate for 1 min and visualized.

### Real-time PCR analysis

The cells were cultured in a 6-well plate for 24 h, then incubated with different ratios of CIK (10:1 or 20:1) for 72 h, and digested, resuspended and lysed. The total mRNA was extracted and reverse transcribed. The transcription levels of MDR1, MRP1 and GST-π, were detected by semiquantitative real-time PCR using the icycler iQ detection system (Bio-Rad, Hercules, CA, USA). The PCR conditions were as follows: Decontamination at 50°C for 60 sec, then denaturation at 95°C for 40 sec, followed by 40 cycles at 95°C for 20 sec and hybridization at 95°C for 30 sec. The oligonucleotide sequences were as follows: MDR1 forward, 5′-AAA AAGATCAACTCGTACCACTC-3′, and reverse, 5′-GCACAAAATACACCAACAA-3′; MRP1 forward, 5′-ACTTCCACATCTGCTTCGTCAGTG-3′ and reverse, 5′-ATTCAGCCACAGGAGGTAGAGAGC-3′; GST-π forward, 5′-TGGGCATCTGAAGCCTTTTG-3′ and reverse, 5′GATCTGGTCACCCAC GATGAA-3′; Bcl-2 forward, 5′-ACGGGGTGAACTGGGGGAGGA-3′ and reverse, 5′-TGTTTGGGGCAGGCATGTTGACTT-3′; and Survivin forward, 5′-AGAACTGGCCCTTCTTGGAGG-3′ and reverse, 5′-CTTTTTATGTTCCTCTATGGGGTC-3′. GAPDH was used for normalization: Forward, 5′-AACTTTGGCATTGTGGAAGG-3′ and reverse, 5′-ACACATTGGGGGTAGGAACA-3′.

### Reporter gene assay

The cells were cultured in a 6-well plate for 24 h and 0.1 μg pGL 4.32[luc2P/NF-kB-RE/Hypro] and pGL 4.32[luc2P/AP-1-RE/Hypro] plasmids were transfected by Lipofectamine 2000 for 6 h. Next, the cells were incubated with different ratios of CIK (10:1 or 20:1) for 72 h, and luciferase activity was measured the by the Dual-Glo Luciferase assay system (Promega Corporation), according to the manufacturer’s instructions, using a Spectra Max M5 microplate reader.

### Statistical analysis

All data are expressed as the mean ± standard deviation. Statistical analysis was performed using SPSS 12.0 (SPSS, Inc., Chicago, IL, USA). The differences between repeated experiments in the three groups were computed using the Student’s t-test and F test. P<0.05 was considered to indicate a statistically significant difference.

## Results

### CIK cell cytokine secretion

**Figure 1:**
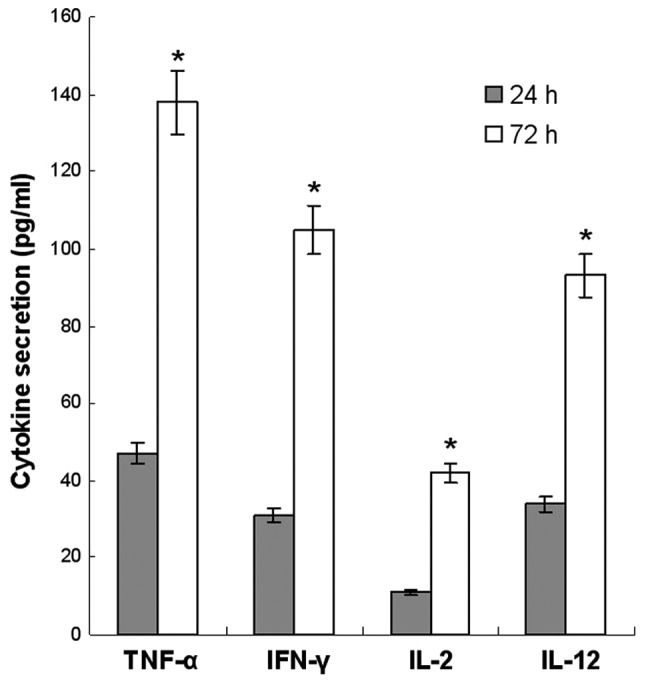
Cytokine secretion of cytokine-induced killer (CIK) cells. The cytokine secretion of CIK cells after 24 and 72 h was analyzed by ELISA assay. The data are presented as the mean ± standard deviation (n=5). Bars indicate the standard deviation. ^*^P<0.05 vs 24 h group. TNF-α, tumor necrosis factor-α; IFN-γ, interferon-γ; IL, interleukin.

Cytokine secretion by the CIK cells was detected by ELISA assay. The CIK cells mainly produced IFN-γ, TNF-α, IL-2 and IL-12 at the end of the culture period. There was an increased secretion of these cytokines following 72 h compared with 24 h (Fig. 1).

### Effect and cytotoxic activity of CIK cells on MDR reversal in K562/ADR and K562 cells

**Figure 2:**
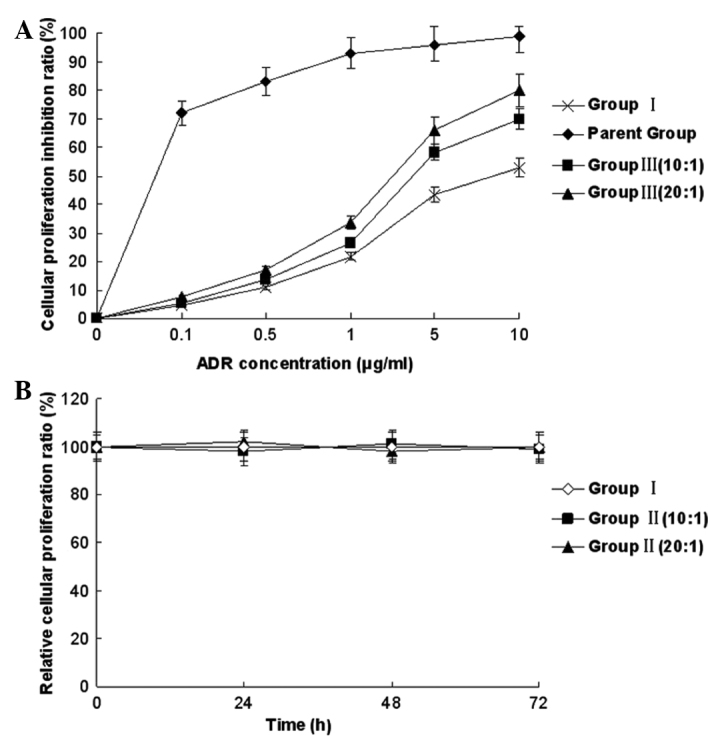
Effect of CIK cells on multidrug-resistance (MDR) reversal and cytotoxic activity in K562/ADR cells. (A) The MDR reversal effect of cytokine-induced killer (CIK) cells on the K562/ADR and K562 cells was measured by MTS assay. (B) The relative cellular proliferation inhibition effect of the CIK cells on the K562/ADR cells was measured by MTS assay. The data are presented as the mean ± standard deviation (n=5). Bars indicate the standard deviation. ADR, Adriamycin.

The cytotoxicity of the CIK cells was evaluated against the K562/ADR and K5462 cell lines by MTS assay. With the different ratio of effector and target cells, the cytotoxic activity was determined. The cytotoxicity of group III at the different ratios of E/T was higher than that of group I, indicating the MDR reversal effect of CIK on the K562/ADR cells. Meanwhile, the cytotoxicity of group II without ADR treatment was not significantly changed compared with that of group I without ADR treatment for 72 h, indicating that there was no significant inhibitory effect of CIK on the K562/ADR cells (Fig. 2).

### CIK-induced apoptosis of K562/ADR cells

**Figure 3:**
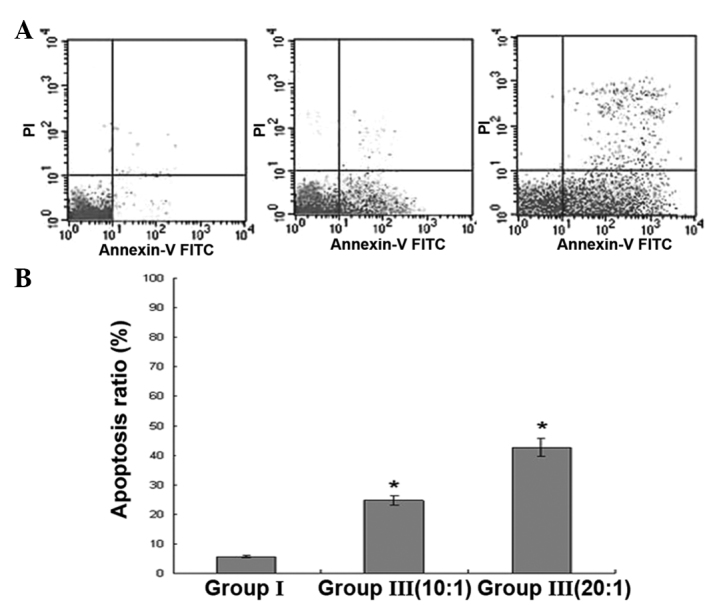
Effect of cytokine-induced killer (CIK) cells on apoptosis induction in K562/ADR cells. (A) The effect of the CIK cells on apoptosis induction in the K562/ADR cells. (B) The data are presented as the mean ± standard deviation (n=3). Bars indicate the standard deviation. ^*^P<0.05 vs. group I. ADR, Adriamycin.

Apoptosis was detected by dual staining with Annexin V-FITC and PI. The apoptosis rate of group III at the different ratios of E/T was higher than that of group I. This revealed that the increased apoptosis observed in group III could be induced by the effect of CIK (Fig. 3).

### Intracellular content of Rh-123 and expression of P-gp and GSH in K562/ADR cells

**Figure 4:**
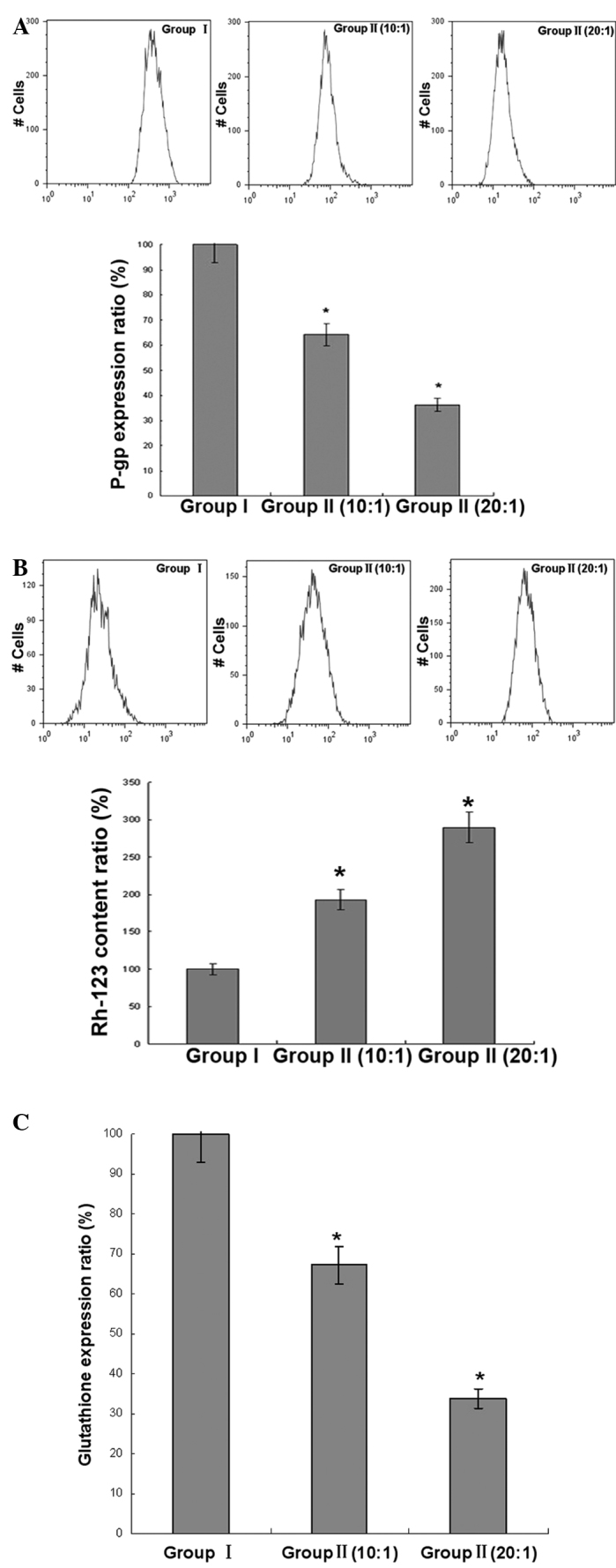
Effect of cytokine-induced killer (CIK) cells on intracellular content of Rh-123 and expression of P-glycoprotein (P-gp) and glutathione (GHS) in K562/ADR cells. (A) The effect of CIK cells on P-gp expression in the K562/ADR cells. (B) The effect of CIK cells on the intracellular Rh-123 content in the K562/ADR cells. (C) The effect of CIK cells on glutathione expression in K562/ADR cells. The data are presented as the mean ± standard deviation (n=3). The bars indicate the standard deviation. ^*^P<0.05 vs. group I. ADR, Adriamycin.

The intracellular Rh-123 content and expression of P-gp was analyzed by flow cytometry. With the different ratios of E/T, the intracellular Rh-123 content of group II was increased compared with group I, and the expression of P-gp and GSH in group II was lower than that of group I, indicating the increased effect of the intracellular Rh-123 content of CIK and the inhibitory effect of the expression of P-gp and GSH of CIK on the K562/ADR cells (Fig. 4).

### Expression of MDR-related gene in K562/ADR cells

**Figure 5:**
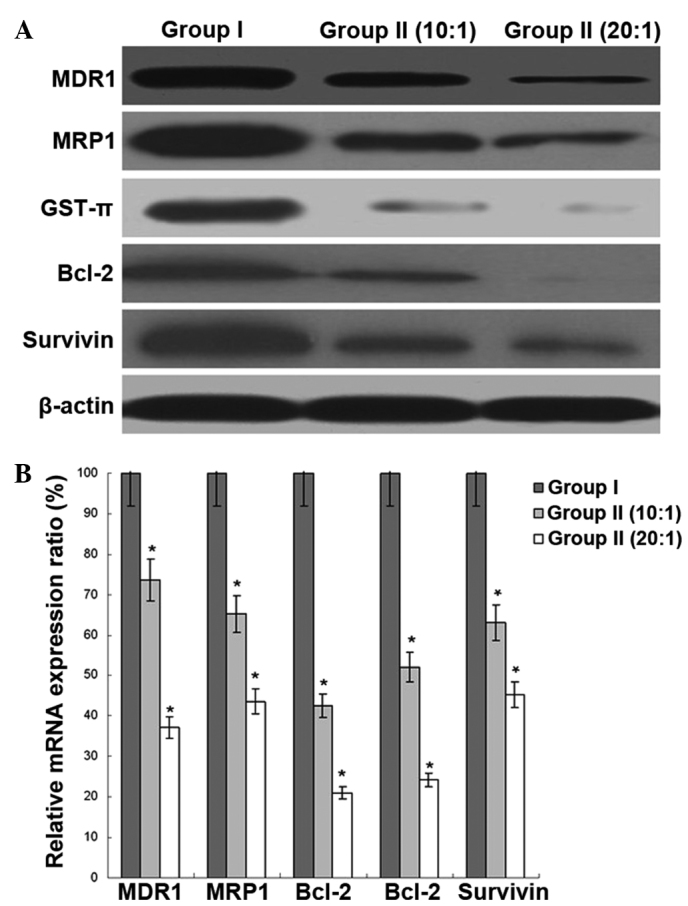
Effect of cytokine-induced killer (CIK) cells on multidrug-resistance (MDR)-related gene expression in K562/ADR cells. (A) The protein expression of MDR gene 1 (MDR1), MDR-associated protein 1 (MRP1), GSH S-transferase-π (GST-π), B-cell lymphoma 2 (Bcl-2) and Survivin was measured by western blot assays in K562/ADR cells. β-actin was an internal reference. (B) The mRNA expression of MDR1, MRP1, GST-π, Bcl-2 and Survivin was measured by real-time PCR assays in K562/ADR cells. GAPDH was an internal reference. The data was presented as the mean ± SD, n=5, bars indicate SD, ^*^P<0.05 vs. Group I. ADR, Adriamycin.

The expression of MDR1, MRP1, GST-π, Bcl-2 and Survivin was analyzed by western blot and real-time PCR assays. With the different ratios of E/T, the protein and mRNA expression of these gene in group II was lower than that of group I, indicating the inhibitory effect of the MDR-related gene expression of CIK on the K562/ADR cells (Fig. 5).

### Regulation of signal transduction molecules in K562/ADR cells

**Figure 6:**
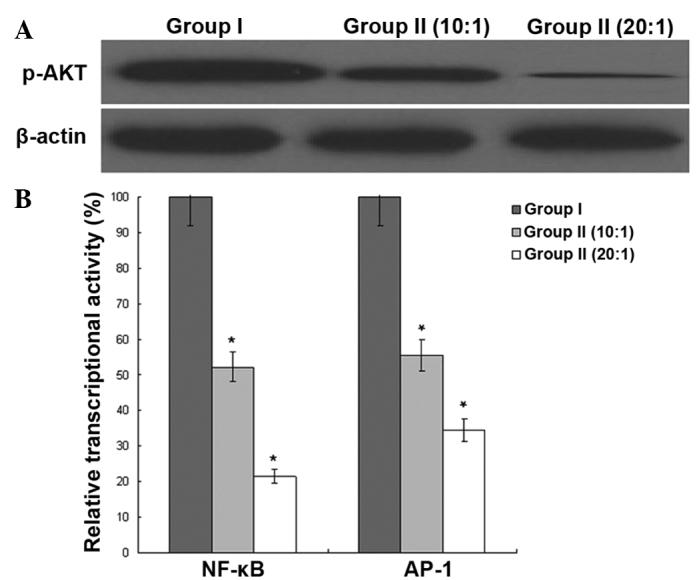
Effect of cytokine-induced killer (CIK) cells on signal transduction molecules regulation in K562/ADR cells. (A) The phosphorylation of AKT was measured by western blot assays in K562/ADR cells. β-actin was an internal reference. (B) The transcriptional activity of nulear factor (NF)-κB and activator protein (AP)-1 was measured by reporter gene assays in K562/ADR cells. The data are presented as the mean ± standard deviation (n=5). The bars indicate the standard deviation. ^*^P<0.05 vs. Group I. ADR, Adriamycin.

The phosphorylation of AKT was analyzed by western blot assay, and the transcriptional activity of nuclear factor (NF)-κB and activator protein 1 (AP-1) was analyzed by reporter gene assay. With the different ratios of E/T, the phosphorylation of AKT and the transcriptional activity of NF-κB and AP-1 of group II were lower than that of group I, indicating the regulative effect of the signal transduction molecules of CIK on the K562/ADR cells (Fig. 6).

## Discussion

Although allogeneic HSCT is a highly effective treatment for leukemia, its therapeutic potential is counterbalanced by treatment-related toxicity and GVHD (5). MDR is the major complication and a formidable obstacle in the therapy of AL (6). Certain previous studies that used cyclosporine and verapamil to reverse the MDR of AL were not practical in the clinic due to the side-effects. Therefore, the development of therapy for the absolute depletion of residual leukemic cells is extremely important. In the present study, CIK was acquired from the peripheral blood of healthy donors, and the K562/ADR cells were cultured with Adriamycin following incubation with CIK. It has been reported that MDR1, MRP1, GST-π, Bcl-2 and Survivin are significant genes with respect to MDR in tumors (7–9). The present study detected that the cytotoxicity, intracellular Rh-123 content and apoptosis rate of the K562/ADR cells was increased, while the expression of P-gp, MDR1, MRP1, GST-π, Bcl-2 and Survivin was decreased by CIK co-cultured with Adriamycin.

The CIK cells have anticancer activity *in vitro* and *in vivo* by the production of effector cytokines, such as IFN-γ, TNF-α, IL-2 and IL-12, which are involved in immunoregulation (10). In the present study, the pattern of cytokines in CIK cells was characterized, including phenotype, cytokine secretion, cytotoxic effects to K562/ADR cells. It was found that a lower E/T ratio (10:1 and 20:1) could induce apoptosis and decrease the expression of MDR-related genes in K562/ADR cells, although there was no significant cytotoxicity with this ratio.

The phosphorylation of AKT could lead to the transcriptional activity of NF-κB and AP-1 (11–13). The present study found that CIK treatment led to downregulated AKT phosphorylation, which meant that the PI3K/AKT pathway was modulated by CIK. Meanwhile, further analysis confirmed that the transcriptional activity of NF-κB and AP-1 was inhibited by CIK treatment.

In summary, the present study provides evidence that CIK has immunoregulatory and other functions in K562/ADR cells, inducing the downregulation of the expression of MDR-related genes and inducing apoptosis of the target cells. Overall, these properties of CIK cells may be beneficial in the treatment of leukemia, particularly for residual AL cells. The study conclusions may provide useful tools for reversing the MDR of AL and be practical in clinical application.
